# P-2042. Development and Maintenance of a Pharmacist-Run COVID-19 Outpatient Treatment Clinic at a Veterans Affairs Hospital

**DOI:** 10.1093/ofid/ofae631.2198

**Published:** 2025-01-29

**Authors:** Sidorela Gllava, Tiffany Ward, Sung Soo Kim, Vivian Vega

**Affiliations:** James A. Haley Veterans' Hospital, Tampa, Florida; James A. Haley Veterans' Hospital, Tampa, Florida; James A. Haley Veterans' Hospital, Tampa, Florida; James A. Haley Veterans' Hospital, Tampa, Florida

## Abstract

**Background:**

In December 2021, oral nirmatrelvir/ritonavir and molnupiravir were approved under Emergency Use Authorization for the treatment of COVID-19 in high-risk outpatients. At that time, National Institutes of Health also recommended intravenous (IV) remdesivir and monoclonal antibodies. The Infectious Diseases team at our hospital designed a protocol for a multidisciplinary Outpatient Clinic that would offer oral and IV therapy to high-risk patients. This protocol used an elaborate scoring guide to identify and prioritize patients based on degree of immune compromise, age, comorbid conditions, and vaccination status.

This clinic made it possible for numerous patients to have access to potentially life-saving therapies. This joint initiative and multidisciplinary effort is, to our knowledge, the first ever Covid Outpatient Clinic, functional 7 days a week/365 days a year.

**Methods:**

Per protocol, if a patient tested positive for COVID-19 and deemed high-risk, a provider would place a consult for outpatient therapy. A pharmacist would then review for the appropriate medication based on symptom onset, renal/hepatic function, and drug-drug interactions (DDI). If a patient was not a candidate for oral therapy, they would be scheduled for an IV infusion. Patients were counseled on adverse effects, DDI mitigation, etc. All consults were completed within 24 hours.

**Results:**

Since January 2022, over 2000 consults were reviewed and 1767 approved for therapy. A total of 1476 patients were treated with oral agents and 291 patients with an IV infusion. Each consult took approximately 45 minutes for pharmacy review and ∼3 hours of nursing time for an infusion. A sub-analysis of 450 nirmatrelvir/ritonavir patients revealed that 65 required a dose adjustment based on renal function and 369 patients required *at least* one adjustment to concomitant medications due to DDIs.

**Conclusion:**

After two years and despite many logistical challenges, we have been able to successfully sustain an Outpatient clinic that provides IV and oral therapy to our veterans, any day of the year. Through this pioneer effort, we have created many foundational interprofessional relationships and we believe this clinic can serve as a model for future endeavors to improve patient care.

**Disclosures:**

All Authors: No reported disclosures

